# Edible Seaweeds

**Published:** 1857-04

**Authors:** 


### MISCELLANEA.

### EDIBLE SEAWEEDS.

It is well known that on different parts of our coasts certain seaweeds are employed by the inhabitants as articles of diet. Some, as the laver (*porphyra laciniata*), is considered so great a delicacy by Devonshire people, that it finds its way even to the *marchands de comestibles* of Bond Street and Piccadilly. Dr. John Davy has recently, in conjunction with Dr. Apjohn,* investigated the properties of some seaweeds. The results at which these inquirers have arrived are deserving of attention, and should be generally known. Their experiments have been directed to determining the esculent properties of carrigeen moss (*chondrus crispus*), dulse or dylisk (*rhodomenia palmata*), laver

* See the Edinburgh New Philosophical Journal, July 1856.

(*porphyra laciniata*), tangle (*laminaria digitata*), and bladder-wrack (*fucus vesiculosus*).

The following is a brief summary of the conclusions arrived at by Dr. Davy :—

The algæ mentioned contain no starch, sugar, or oily matter. Chondrus crispus contains a large amount of gummy and gelatinous matter ; as does also the rhodomenia palmata, and the porphyra laciniata. Very little gum, and no gelatine, were discovered in the laminaria digitata ; and only a trace of gelatinous matter appears to have been obtained from the bladderwrack. All contain more or less iodine, which is removed by maceration or bleaching. In one instance, Dr. Davy observes, the seaweed was deprived (by washing) not only of adhering saline matter, but also of a portion of its own substance, and of the iodine entering into the composition of the algæ, thereby necessarily deteriorating its value. This remark is specially applicable to chondrus crispus, which is largely exported from Ireland in a bleached state. Even the nitrogenous constituents appear to be diminished by the bleaching process. The point of chief interest, however, is the demonstration afforded by Dr. Apjohn, of the large amount of nitrogen contained in some seaweeds, which justifies the reputation they have amongst the poor, to whom their use as an article of food is chiefly restricted. The following is a tabulated account of Dr. Apjohn's experiments :—

*Account of Dr. Apjohn's Experiments.*

| | Water. | Dry Matter. | Per cent. of Nitrogen in Dry Matter. | Protein contained in Dry Matter. |
|---|---|---|---|---|
| *Chondrus crispus*, bleached ...... | 17·92 | 82·08 | 1·534 | 9·587 |
| „ *crispus*, unbleached .... | 21·47 | 78·53 | 2·142 | 13·387 |
| *Gigartina mammillosa* .......... | 21·55 | 78·45 | 2·198 | 13·737 |
| *Chondrus crispus*, bleached ...... | 19·79 | 80·21 | 1·485 | 9·281 |
| „ *crispus*, unbleached .... | 19·96 | 80·04 | 2·510 | 15·687 |
| *Laminaria digitata*, or Dulse Tangle | 21·38 | 78·62 | 1·588 | 9·925 |
| „ *digitata*, or Black Tangle | 31·05 | 68·95 | 1·396 | 8·725 |
| *Rhodomenia palmata*, or Dylisk ... | 16·56 | 83·44 | 3·465 | 21·656 |
| *Porphyra laciniata*, or Laver ..... | 17·41 | 82·59 | 4·650 | 29·062 |
| *Iridæa edulis* .................. | 19·61 | 80·39 | 3·088 | 19·300 |
| *Alaria esculenta*, or Murlins ...... | 17·91 | 82·09 | 2·424 | 15·150 |
| Means ....... | 20·42 | 79·58 | 2·407 | 15·045 |

The percentage of nitrogen found in various other edible substances is given by way of comparison, thus :—

| | | N. |
|---|---|---|
| Potatoes, contain | . . . . . . . | ·541 |
| Flour of first quality | . . . . . . . | 1·817 |
| Beet roots (Mean of 13 experiments) | . . | 1·848 |
| Mangolds (Mean of 3 experiments) | . . . | 1·781 |
| Swedish turnips (Mean of 5 experiments) | . . | 1·843 |
| Means | . . . | 1·567 |

It follows from these two series that the proportion of nitrogen contained in these algæ exceeds not only that of the ordinary articles of vegetable food, but even that of wheaten flour, being as 2·407 to 1·817.

Dr. Davy suggests, further, that the iodine and bromine contained in these esculent seaweeds render them worthy of more extended use; he states that where they are commonly employed, bronchocele is unknown, and scrofulous complaints and pulmonary consumption are rare. Apart from their culinary importance, seaweeds are in great repute as manures, wherever they are easily obtained. This is accounted for by their wealth in nitrogen, and in those inorganic substances which are essential to our cereals, especially phosphate and carbonate of lime, and one or both of the fixed alkalies. In the economy of nature, these marine plants may be regarded as exercising no less important a part than terrestrial plants. "May they not," says Dr. Davy, "be considered as purifiers of sea-water, tending always to remove excess of carbonic acid, and probably of azote? and may they not be viewed as the restorers (they certainly are the collectors) of substances possessing excellent medicinal qualities, viz., iodine and bromine, which so specially belong to them, and, in them, seem providentially saved and stored up for the use of man."

We cannot but regard this inquiry as one fraught with much interest of a scientific and a practical character. We are, therefore, much gratified to learn that two prizes of £50 and £20 respectively are now offered for the best approved essays on the application of the marine algæ and their products, as food or medicine for man and domestic animals (cattle, sheep, etc.) Competitors are required to give the results of their original investigations on seaweeds, especially on the chemistry of their nutrient principles, and must therefore prepare a series of specimens, illustrative of the best mode of collecting, preserving, and transporting, in a state fit for food, the nutritive species. It is proposed that the prizes shall be awarded by the following gentlemen:—T. C. Archer, Esq., Liverpool; Professor Balfour, Edinburgh; Dr. John Davy, Ambleside; Professor Graham, Royal Mint, London; Dr. Greville, Edinburgh;

Sir W. J. Hooker, Kew; Dr. Lindsay, Perth; Sir W. C. Trevelyan, Bart., Wallington; and Professor George Wilson, Edinburgh.

